# GENDER DIFFERENCES IN CAREGIVER BURDEN AMONG FAMILY CAREGIVERS OF PERSONS WITH DEMENTIA: A SYSTEMATIC REVIEW

**DOI:** 10.1093/geroni/igad104.1386

**Published:** 2023-12-21

**Authors:** Thitinan Duangjina, Thanakrit Jeamjitvibool, Chang Park, Rebecca Raszewski, Valerie Gruss, Cynthia Fritschi

**Affiliations:** College of Nursing, the University of Illinois Chicago, Chicago, Illinois, United States; College of Nursing, the University of Illinois Chicago, Chicago, Illinois, United States; College of Nursing, the University of Illinois Chicago, Chicago, Illinois, United States; The University of Illinois Chicago, Chicago, Illinois, United States; College of Nursing, the University of Illinois Chicago, Chicago, Illinois, United States; UIC College of Nursing, Chicago, Illinois, United States

## Abstract

Family caregiving for individuals with dementia is associated with high levels of burden. Caregiver burden is likely contextual and related to sociocultural norms, including those for gender. We conducted a systematic review and meta-analysis to examine gender differences in caregiver burden among family caregivers of persons with dementia and to compare such gender differences across cultures. After searching five databases—CINAHL, Medline/PubMed, EMBASE, PsycINFO, and Sociological Abstracts, we included relevant articles published in English through December 2022. Two independent reviewers extracted study data and assessed the risk of bias. Forty-three studies were included, and from those, 17 effect sizes were extracted for meta-analysis. We found that family caregivers providing homecare were mainly female and adult children of care recipients. Most studies used the Zarit Burden Interview to measure caregiver burden. Random-effects models revealed moderate effects of gender, with higher burden in female than in male family caregivers (d = 0.41, 95% confidence interval: 0.32 to 0.50, p<.05). However, we found that only 20% of Asian studies reported effects of gender on caregiver burden, while more than half the U.S. (83.33%) and European (56.41%) studies reported gender effects. We conclude that, due to sociocultural norms, female Asian caregivers may more readily accept their caregiving role than females in other regions. Thus, future studies should include measures of cultural expectations related to caregiving. Also, researchers should more extensively explore gender-specific sources of caregiver burden before creating gender-specific caregiver interventions and social policy recommendations for alleviating the burden of female caregivers.

